# Tuberculosis Presenting With Acute Myocarditis and Systolic Heart Failure

**DOI:** 10.7759/cureus.13229

**Published:** 2021-02-08

**Authors:** Nikhil Choudhary, Hailu Abera, Ranajit B Naik

**Affiliations:** 1 Cardiology, Narayana Multispeciality Hospital, Jaipur, IND; 2 Medicine, St. Paul’s Hospital Millennium Medical College, Addis Ababa, ETH; 3 Cardiothoracic Surgery, Narayana Multispeciality Hospital, Jaipur, IND

**Keywords:** myocarditis, systolic heart failure, tuberculosis

## Abstract

Tuberculosis presenting with myocarditis and severe systolic dysfunction is rarely reported. So far, only a few cases were reported from India. Our aim is to report this rare presentation of a common disease that we encountered at Narayana Multispecialty Hospital, Jaipur. A 34-year-old lady having disseminated tuberculosis involving lung, lymph node, and myocardium with severe left ventricular systolic dysfunction received medical treatment in our hospital. She had elevated cardiac biomarkers, severe left ventricular regional wall hypokinesis with an ejection fraction of 25-30%, bilateral upper and middle lobe ground-glass opacities, as well as mediastinal and hilar lymphadenopathies on chest computed tomography scan and normal coronary angiogram. The patient was started with anti-tuberculosis therapy, a beta-blocker, an angiotensin-converting enzyme inhibitor, and a corticosteroid and discharged after one week in a stable condition. The third month of follow up showed recovery and improvement in cardiac function.

## Introduction

Tuberculosis (TB) involving myocardium is very rare, and its prevalence was reported to range between 0.14% and 0.2% in several studies [1,2]. The diagnosis of isolated tuberculous myocarditis is very difficult and usually made at autopsy. However, concomitant pulmonary tuberculosis is present in half of the reported cases. Tuberculous myocarditis is mostly asymptomatic but may present acutely with congestive heart failure, ventricular fibrillation, long QT syndrome, dilated cardiomyopathy, and even sudden cardiac arrest [3]. In this article, we describe the clinical presentation, diagnostic workup, and treatment given for our case and briefly review the current literature.

## Case presentation

A 34-year-old Indian woman presented at the Emergency Department of Narayana Multispecialty Hospital (Jaipur, India) in September 2017 with low-grade fever, night sweating, cough, and progressive shortness of breath of one-month duration. The cough was initially productive of whitish sputum and, since two days, had become blood mixed. She had no chest pain, orthopnea, or paroxysmal nocturnal dyspnea. There was no history of diabetes mellitus, hypertension, smoking, illicit drug use, or renal disease. There was no history of tuberculosis or contact with a patient with a chronic or longstanding cough.

**Figure 1:**
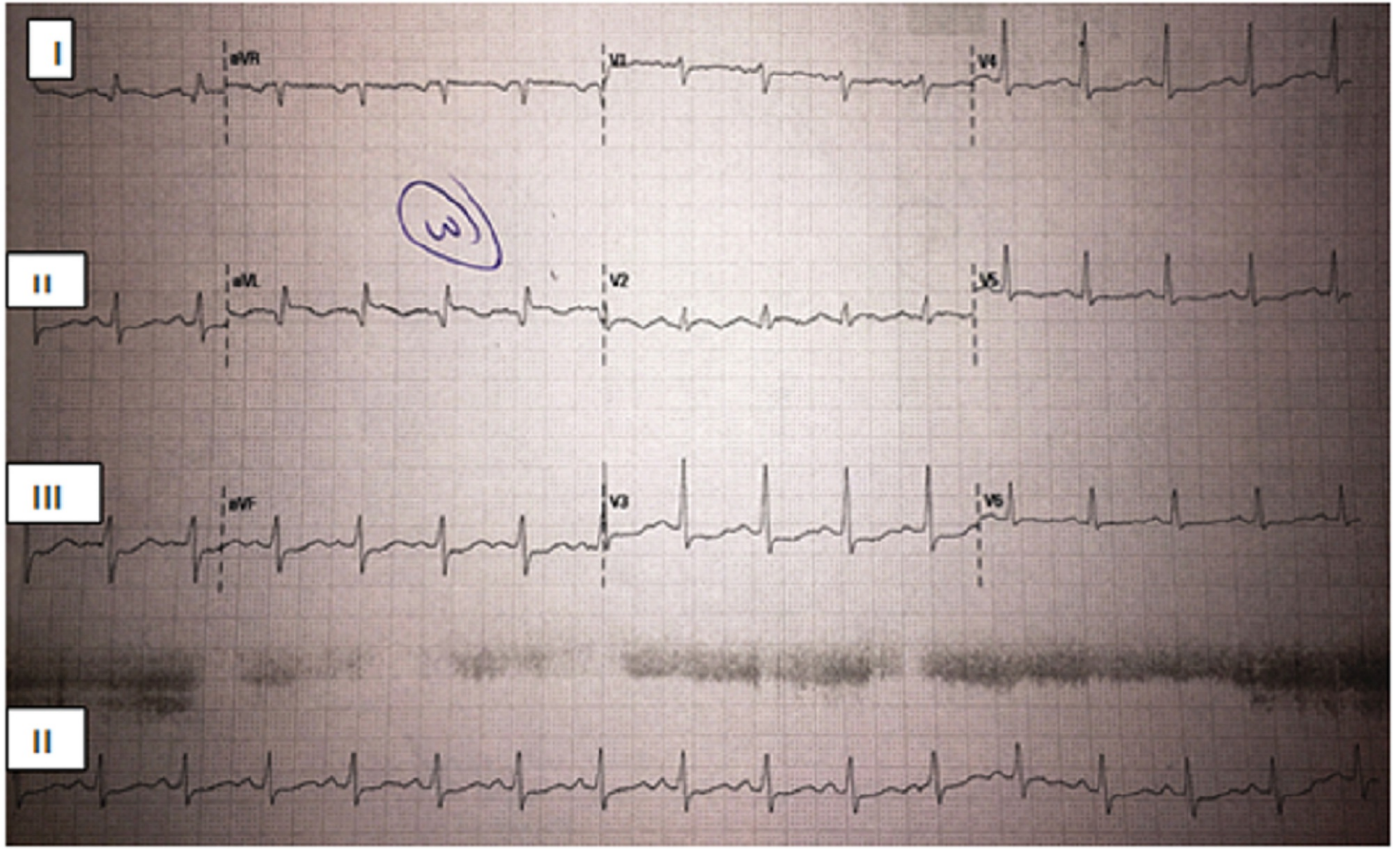
An electrocardiogram revealed sinus rhythm, ventricular rate of 104 beats per minute, and T-wave inversion on leads I and aVL

On examination, the patient was comfortable having stable vital signs. There was scattered fine crepitation in both lung fields and no remarkable finding in other systems. Laboratory workup revealed hemoglobin 9.3 mg/dl, white blood cells 4200/mm3, platelets 432,000/mm3, mean corpuscular volume (MCV) 87 fl, erythrocyte sedimentation rate 95 mm/1st hour, and random blood sugar 99 mg/dl. Renal function tests and electrolytes were normal. Liver function tests showed aspartate transaminase 174 mg/dl, alanine transaminase 84 mg/dl, and alkaline phosphatase 209 mg/dl. Serum albumin, bilirubin, and coagulation panel were normal. Serologic tests for hepatitis B virus, hepatitis C virus, and HIV were negative. Thyroid-stimulating hormone, antinuclear antibody, and serum procalcitonin levels were also normal. The abdominal ultrasound was normal. Cardiac biomarkers were elevated with creatine kinase-MB 24.1 ng/ml, troponin I 4.69 ng/ml, and brain natriuretic peptide 880 pg/ml. Electrocardiography (ECG) showed sinus rhythm, ventricular rate of 104 beats per minute, and T-wave inversion on leads I and aVL, as shown in Figure 1.

**Figure 2:**
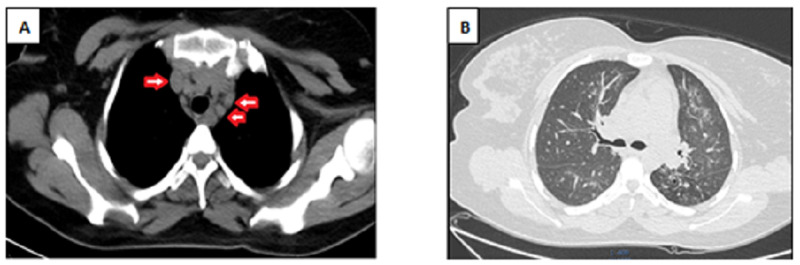
Chest computed tomography showed: (A) Multiple small- and medium-sized discrete non-calcified mediastinal lymphadenopathies (arrow), and (B) Multiple ground glass nodular opacities in both lung fields

Echocardiography showed regional wall motion abnormality with apical septal, apical, and anterolateral wall severe hypokinesis to akinesis. There was also mild mitral regurgitation, mild tricuspid regurgitation, and severe left ventricular systolic dysfunction with an ejection fraction of 25-30%. The pericardium was not thickened, and there was minimal pericardial effusion. A coronary angiogram showed normal coronary arteries and no ischemia or occlusion. Chest CT revealed multiple enlarged small- and medium-sized discrete non-calcified hilar and mediastinal lymph nodes. There were also multiple ground glass nodular opacities in both upper and middle lung fields bilaterally and right lower lung field suggestive of infectious bronchiolitis, likely tuberculosis (Figure 2).

The patient was admitted and managed for systolic heart failure and less hepatotoxic anti-tuberculosis therapy, including isoniazid, moxifloxacin, and ethambutol. She was also started with a corticosteroid (prednisolone). After one week of hospital stay, the patient’s clinical condition improved, including a drop in liver transaminases, and she was discharged. On the third month of follow up, she showed excellent recovery on antituberculosis chemotherapy with marked improvement in cardiac function.

## Discussion

Despite the lack of myocardial biopsy for definitive diagnosis of myocardial involvement in our patient, the clinical presentation, investigations, and dramatic anti-tuberculosis treatment response were all in favor of myocardial dissemination. 

Tuberculous myocarditis is a very rare disease with only a few reported cases in the literature. Cardiac manifestations have been rarely reported in immunocompetent patients with pulmonary tuberculosis. It is estimated that only 1% of all cases of tuberculosis have cardiac involvement [2]. Tuberculosis mostly affects the pericardium in endemic areas; involvement of myocardium leading to severe systolic heart failure is very rare [2,3].

Tuberculous myocarditis results from the spread of Mycobacterium tuberculosis bacilli to myocardium either through hematogenous route or direct extension from pericardium or retrograde lymphatic spread from bronchial and mediastinal lymph nodes due to pulmonary tuberculosis [2,4]. In our patient, it was likely to be through lymphatic spread from pulmonary focus. The majority of reported cases of tuberculous myocarditis are males under 45 years of age. The frequent clinical presentations are congestive heart failure, acute chest pain mimicking myocardial infarction, refractory ventricular tachycardia, long QT syndrome, and sudden cardiac death [3]. The presentation of this case as systolic heart failure is rare because myocardial tuberculosis itself is asymptomatic, rarely diagnosed during life, and diagnosis is usually made after an autopsy study [5].

Accurate diagnosis of tuberculous myocarditis is limited by the difficulties and associated complications in doing an endomyocardial biopsy. However, diagnosis is usually made on the basis of medical history, clinical presentation, laboratory findings, and imaging studies. Myocarditis can be diagnosed clinically in the presence of elevated cardiac markers of injury (cardiac troponin I or troponin T, and creatine kinase-MB), with regional or globally depressed left ventricular function seen on echocardiography or cardiac magnetic resonance scan in the absence of coronary artery disease. Unlike in patients with pericarditis, ECG findings of myocarditis are usually non-specific [6]. In our case, the presence of elevated markers of myocardial injury, left ventricular regional wall hypokinesis with depressed systolic function, and absence of coronary artery disease strongly suggest myocarditis. On the other hand, the dramatic response of her myocardial function to anti-tuberculosis treatment indicates a tuberculous etiology.

The management of tuberculous myocarditis associated with left ventricular systolic dysfunction is mainly aimed at the specific pathogen, preventing further myocardial inflammation and remodeling. Based on the 2013 European Society of Cardiology Working Group on Myocardial and Pericardial Diseases guidelines, patients with inflammatory myocarditis may benefit from immunosuppressive or immunomodulatory therapies, and those with an ejection fraction less than 40% should be treated with guideline-based heart failure therapy [7].

In the present case, the prescribed medications were anti-tuberculosis therapy, carvedilol, ramipril, ivabradine, and prednisolone. After three months of treatment, the patient recovered well and became asymptomatic.

## Conclusions

In conclusion, pulmonary tuberculosis complicated by myocarditis and left ventricular dysfunction has been rarely reported. Early diagnosis and management are associated with a good outcome. Therefore, in tuberculosis endemic areas, it should be suspected in patients with unexplained left ventricular systolic dysfunction in the absence of coronary artery disease.
